# Predictive value of brachial artery flow-mediated dilation on coronary artery abnormality in acute stage of Kawasaki disease

**DOI:** 10.1038/s41598-021-87704-y

**Published:** 2021-04-14

**Authors:** Yizhou Wen, Xianmin Wang, Yonghong Guo, Mei Jin, Jimei Xi, Tingting Chen, Kun Shi, Yaheng Lu

**Affiliations:** 1 Chengdu Women’s and Children’s Central Hospital, School of Medicine, University of Electronic Science and Technology of China, Chengdu, 611731 China; 2 Sichuan Women’s and Children’s Hospital, Women’s and Children’s Hospital Affiliated to Chengdu Medical College, Chengdu, 610045 China

**Keywords:** Cardiology, Diseases, Health care, Medical research

## Abstract

Coronary artery abnormalities (CAAs) are a severe complication of Kawasaki disease (KD) that may lead to cardiovascular events. Given the evidence that brachial artery flow-mediated dilation (FMD) decreases in children after the onset of KD, we hypothesized that it could be an early marker of CAA development in the acute stage and investigated its relationship with variation in the coronary artery diameter. A total of 326 sex- and age-matched children were enrolled, including 120 with KD, 109 febrile children and 97 healthy controls. In this study, FMD was significantly decreased in the KD group compared with the febrile and healthy groups. FMD was lower in the CAA group than in the no coronary artery abnormality group. The comparison of FMD showed an obvious difference among the CAA subgroups. The FMD in the coronary aneurysm (CA) group showed a strong negative correlation with the pretreatment maximum coronary artery Z-score (preZmax). While preZmax was 2.5, the receiver operating characteristic curve indicated an optimal cutoff point of 3.44% for FMD. FMD ≤ 3.44% could be considered as a signal of coronary lesions in acute stage of KD.

## Introduction

Kawasaki disease (KD) is a self-limiting systemic vasculitis of small- and medium-sized arteries. With its high incidence of inflammatory dilation and infiltration in the coronary arteries, KD has become the most common cause of childhoodacquired heart disease^1^. As a noninvasive measurement of endothelial function, brachial artery flow-mediated dilation (FMD) decreases in each phase of KD and remains low for a long time, which suggests sclerosis during the convalescent phase^2,3^. The Z-score of the coronary artery may reflect the presence of coronary artery abnormalities (CAAs) in KD more objectively than a direct measurement of the diameter^4,5^. However, the association between endothelial dysfunction and the severity of CAAs in the acute phase of KD remains unclear. This study aimed to determine whether predictable endothelial dysfunction exists even before dilation of the coronary artery by comparing FMD among different groups, as well as whether there is a correlation between FMD and the pretreatment maximum coronary artery Z-score (preZmax), during acute KD.

## Materials and methods

### Study subjects

In this prospective, case–control study, healthy subjects completing regular check-ups and patients presenting with KD or fever at Chengdu Women's and Children's Central Hospital (Chengdu, Sichuan province, China) between May 2017 and June 2019 were screened for participation. The inclusion criteria for the KD group were the American Heart Association (AHA) guidelines^6^ for KD, an age from 0 to 18 years old. The exclusion criteria were as follows: (1) previous or current medical history of dyslipidaemia, diabetes, inherited cardiovascular disease, inherited metabolic disease, cystinuria, chronic kidney disease, blood disease, Kawasaki disease, etc.; and (2) any history of major surgery, blood transfusion, or use of vasoactive agents within 3 months before our examination.

### Data collection

General clinical data, including age, sex and BMI, were extracted from the medical records. The baseline brachial diameter, FMD and preZmax were measured by one study-specific sonographer (J.X.) in a quiet and temperature-controlled condition (25 °C) according to standard methods for ultrasound imaging of coronary arteries in children^6–8^. An ultrasound machine (SAMSUNG UGEOHM70A) with a 7-13L transducer was used. All subjects were asked to sleep late and lay at rest in the supine position for 10 min before the examination and chloral hydrate (10% solution at 0.3–0.5 ml/kg, maximum 10 ml) was used to sedate uncooperative subjects who were ready for ultrasound examination. For young children especially those who woke up or cried during the test, more patience and attempts were required. Incentives such as toys or candies could be adopted if necessary. Children with emotional instability needed additional dose of sedatives especially those who could not be calm down by any kinds of comfort. Sedatives were selected according to the following order: oral first, then rectal, intravenous the last. Parents were asked to hold their children in a fixed position with the help of doctors. FMD was determined with reference to the methods described by Celermajer et al.^9^ The Z-score was calculated using the LMS method described by Kobayashi et al.^8^, respectively. Aforementioned ultrasound machine was used to detect the brachial artery 2–5 cm above the antecubital fossa of the right arm. The distance between the anterior and the posterior intima of the vascular wall at the end of diastole (D1) was measured as a baseline internal diameter through the longitudinal two-dimensional images. Then the brachial artery was occluded by inflating the blood pressure cuff to 50 mmHg above the subject’s resting systolic blood pressure. The cuff remained inflated for 5 min and was then quickly deflated. Sixty seconds later, the diastolic diameter (D2) was obtained again. The value of FMD was calculated using the following formula: FMD = [(D2 − D1)/D1] × 100%. The maximum Z-scores of the left main coronary artery, left anterior descending coronary artery, left circumflex artery and right coronary artery was taken as preZmax. We chose the one with the smaller inner diameter (which may got a heavier dilation) to count when the Z score of any two coronary arteries was the largest. Either LAD or LCX with a bigger Z-score in our statistical process replaced some stubby variations of left main coronary artery that failed to get a normal measurement. The FMD and Z-score in the KD group were measured before medication treatment (intravenous immune globulin (IVIG) therapy^6^ at the beginning of KD). Each parameter was measured three times for an average value as a final result.

### Statistical analysis

Analyses were performed using SPSS 20.0 statistical software. The FMD and Z-score values were analysed using the Kruskal–Wallis H-test and Mann–Whitney *U* test. Other mean values were analysed using ANOVA. Enumeration data were analysed by chi-square test. Spearman’s rank correlation and ROC curve analysis were applied in the analysis of the relationship between FMD and preZmax. *P* < 0.01 was considered statistically significant.

### Ethical approval

The Chengdu Women's and Children's Central Hospital ethics committee approved this study. Clinical informed written consent was obtained from the guardians of each child. All methods were carried out in accordance with the Declaration of Helsinki.

## Results

**Figure 1:**
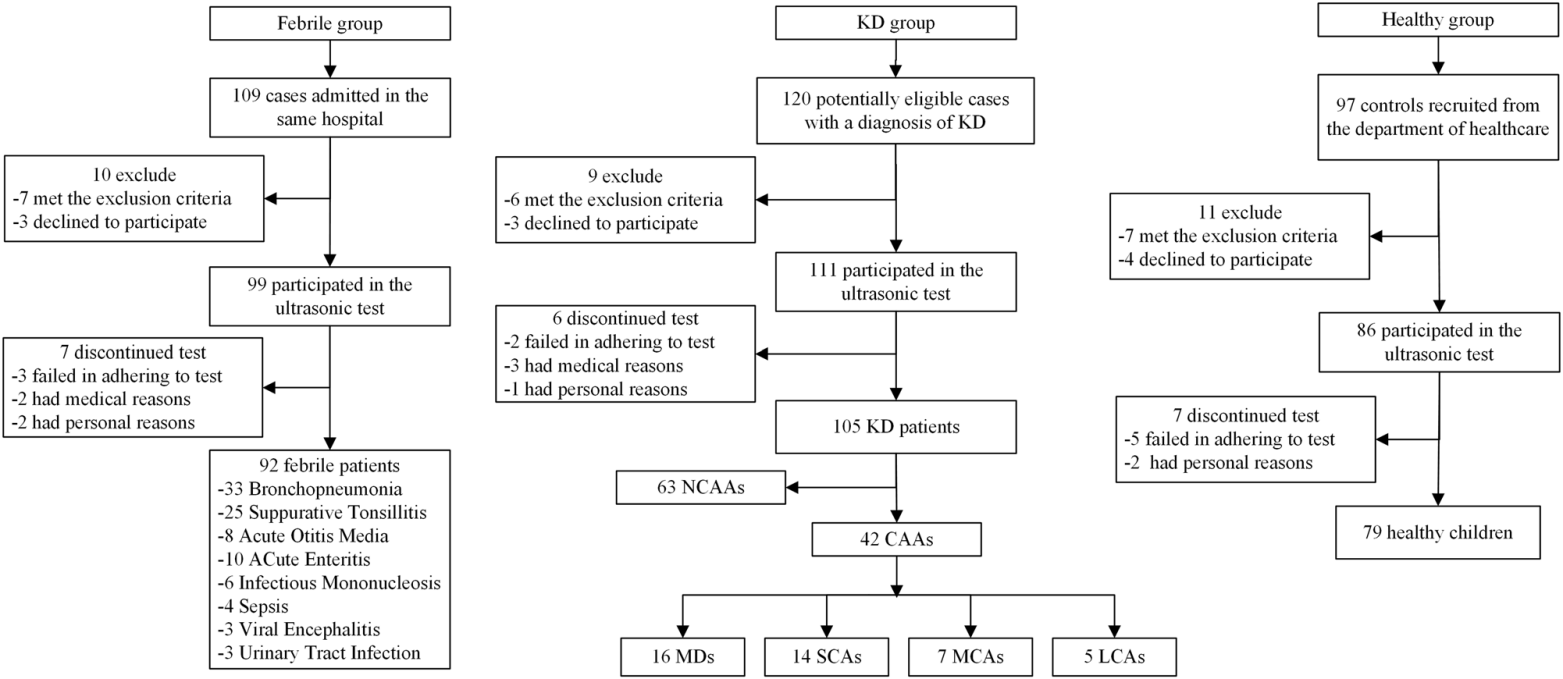
Participant flow diagram.

**Figure 2:**
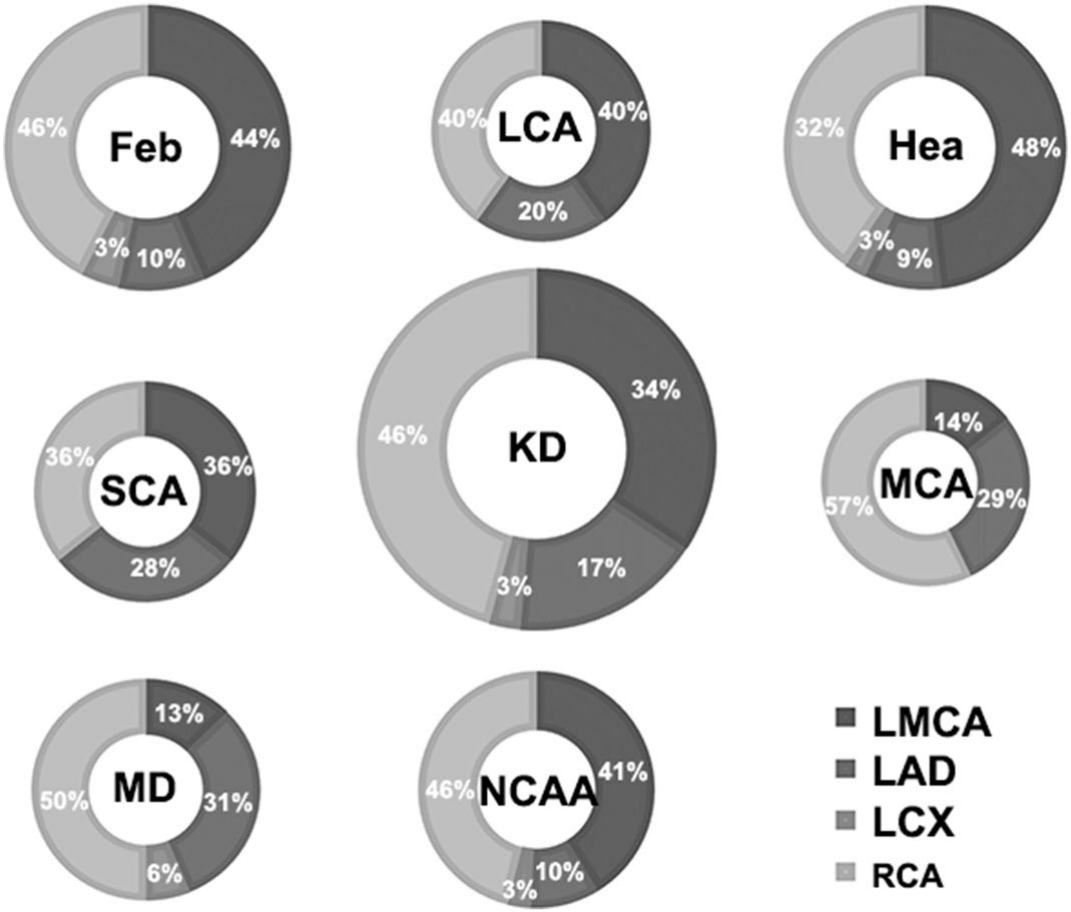
Coronary segment distribution of the preZmax in different groups. The number shows the percentage of coronary segment which the preZmax came from.

**Figure 3:**
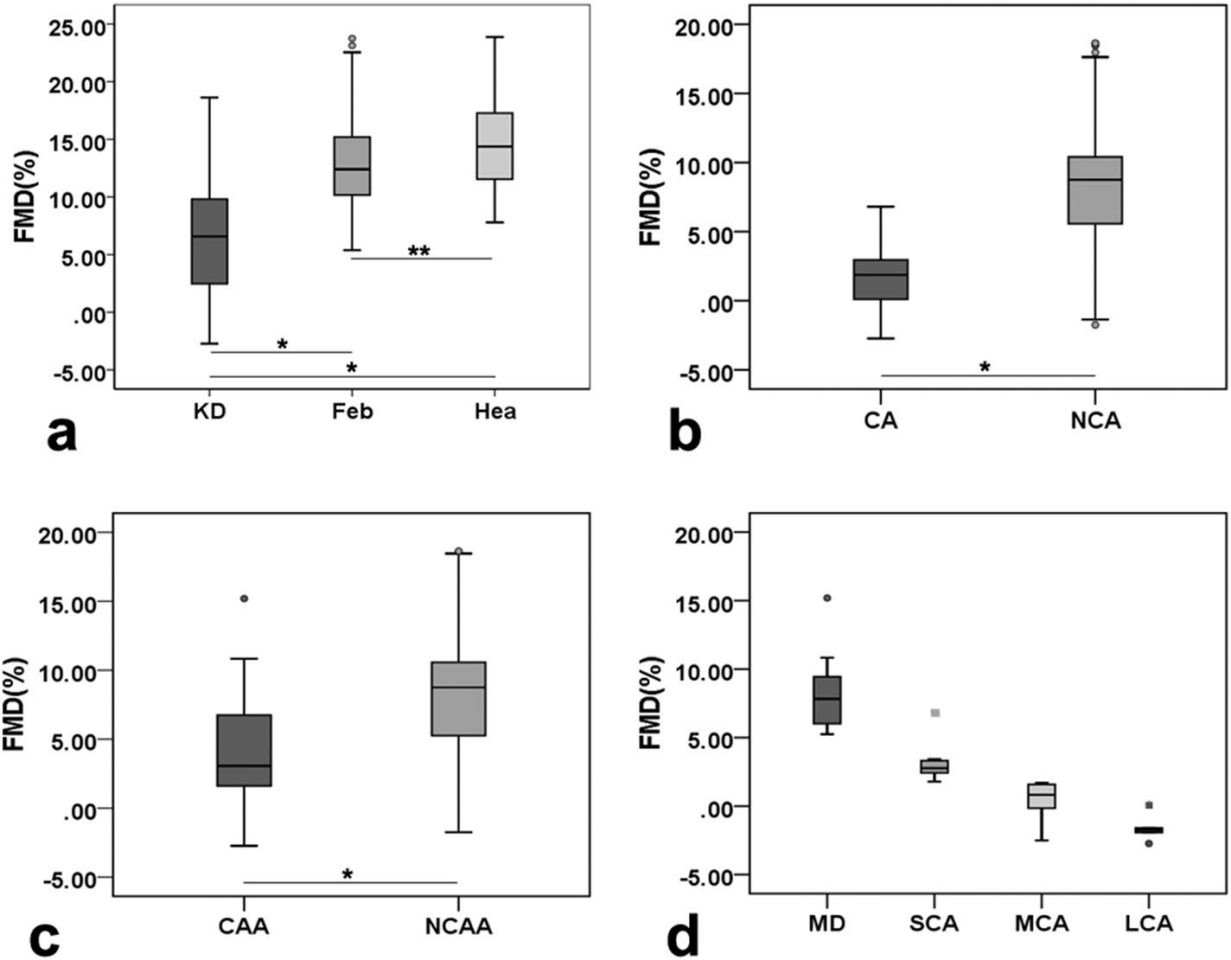
Comparison and distribution of flow-mediated dilation (FMD) among groups. *KD* Kawasaki disease; *Feb* febrile (controls); *Hea* healthy (controls); *CAA* coronary artery abnormality; *NCAA* no coronary artery abnormality; *CA* coronary aneurysm; *NCA* no coronary aneurysm; *MD* mild dilation; *SCA* small coronary aneurysm; *MCA* middle coronary aneurysm; *LCA* large coronary aneurysm. Box plots showing (**a**) comparison of FMD among the KD, febrile and healthy groups; (**b**) comparison of FMD between the CAA and NCAA groups; (**c**) comparison of FMD between the CA and NCA groups; (**d**) distribution of FMD among the subgroups of CAA group. (*P < 0.01; **P > 0.05). Circles denote outlier cases, and squares denote extreme cases.

**Figure 4:**
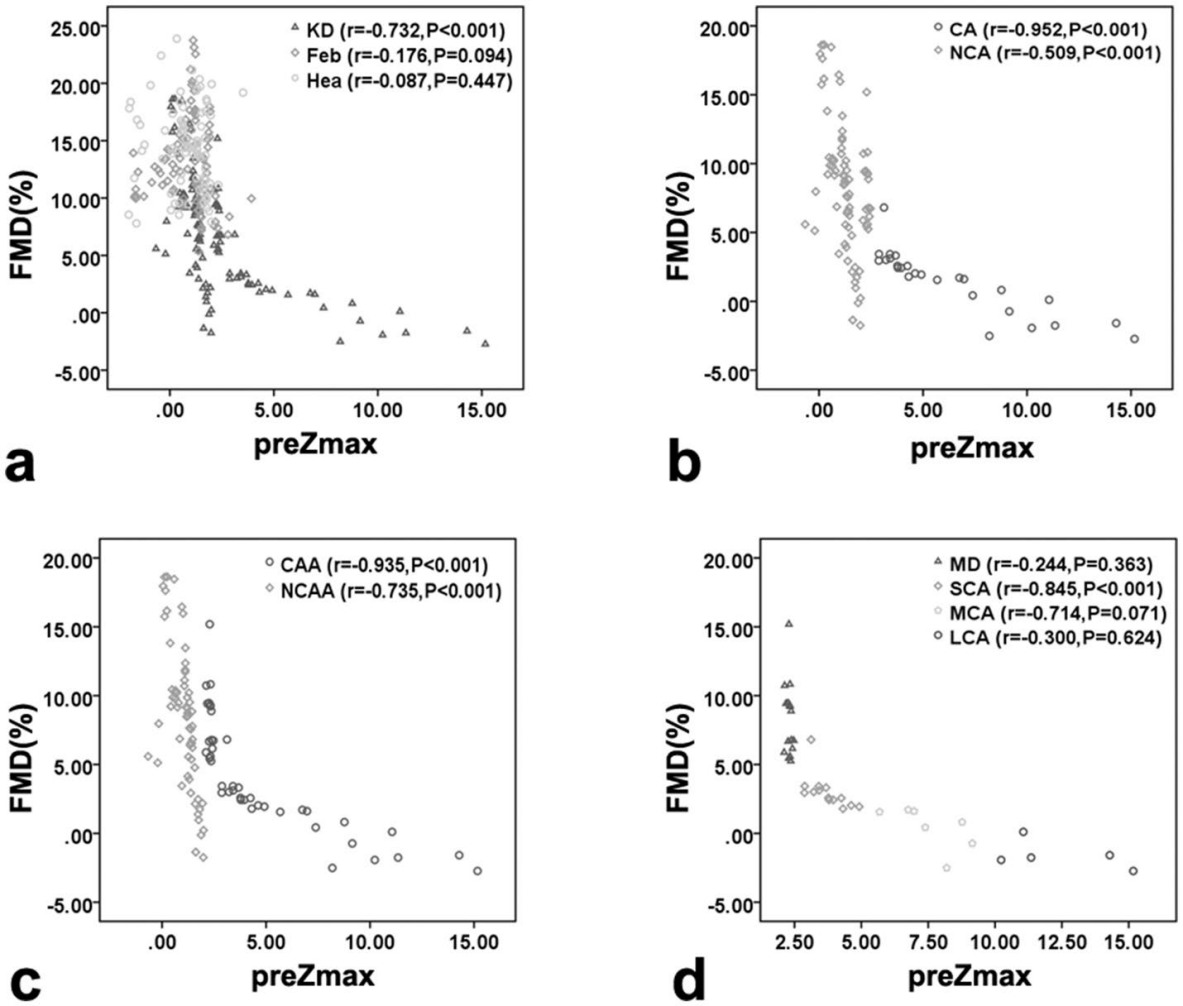
Correlation between flow-mediated dilation (FMD) and pretreatment maximum coronary artery Z-score (preZmax) in groups. *KD* Kawasaki disease; *Feb* febrile (controls); *Hea* healthy (controls); *CA* coronary aneurysm; *NCA* no coronary aneurysm; *CAA* coronary artery abnormality; *NCAA* no coronary artery abnormality; *MD* mild dilation; *SCA* small coronary aneurysm; *MCA* middle coronary aneurysm; *LCA* large coronary aneurysm. Scatter plots showing (**a**) correlation between FMD and preZmax in the KD, febrile and healthy groups; (**b**) correlation between FMD and preZmax in the CA and NCA groups; (**c**) correlation between FMD and preZmax in the CAA and NCAA groups; (**d**) correlation between FMD and preZmax in the CAA subgroups.

**Figure 5:**
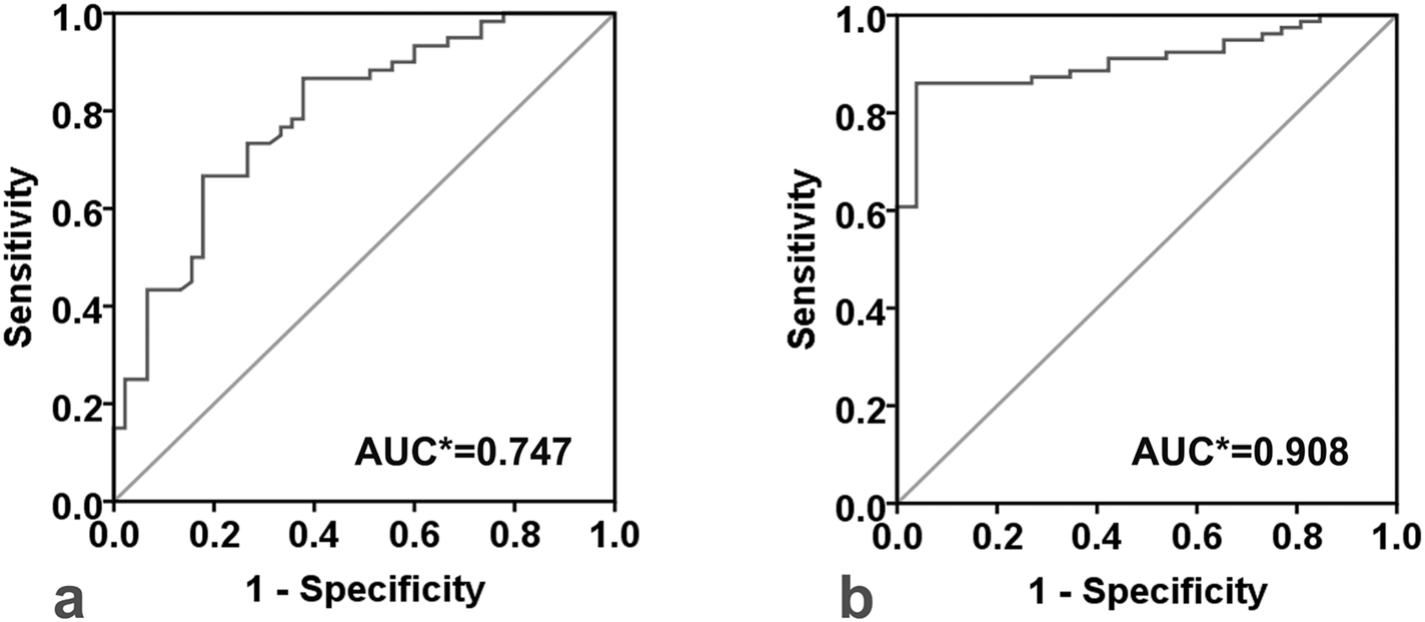
(**a**) Receiver operating characteristic (ROC) curve analysis for flow-mediated dilation (FMD) in predicting coronary dilation (preZmax > 2.0) during acute Kawasaki disease. (**b**) ROC curve analysis for FMD in predicting coronary aneurysm (preZmax ≥ 2.5) during acute Kawasaki disease. AUC* area under the curve.

Ninety-seven healthy subjects completing regular check-ups, 109 febrile children and 120 children with KD were included (Fig. 1). All subjects were randomly selected during the same period at the same hospital mentioned above. Two hundred and ninety six cases participated in the ultrasonic test, while 5 of them quit directly for personal reasons and 5 quit because of medical reasons, both before the test began. Then 286 cases involved in the ultrasonic test progress. The initial failure rate was about 17.8%. In kids who failed at the first try, 15% woke up or cried during the ultrasonic test and the rest 2.8% failed because of an occasional instrument malfunction. As 32.6% of those first-try-failed children could be calm down by comfort, the rest of them need additional dose of sedatives. Kids who could not cooperate to complete the test had to quit, so that the final failure rate was 3.5%. As shown in Table 1, there were no significant differences in baseline characteristics (age, sex, BMI) among the KD, febrile and healthy groups (*P* > 0.05). Coronary segment distribution of preZmax varied in Fig. 2. There were significant differences in FMD among those groups (F = 86.190, *P* < 0.01). The FMD in the KD group was significantly lower than that in both the febrile and healthy groups on pairwise comparison (F =  − 6.298, − 7.843, *P* < 0.01), while no significant difference in the FMD was observed between the febrile and healthy groups (F = 1.546, *P* = 0.025). The FMD in the CAA group was significantly lower than that in the no coronary artery abnormality (NCAA) group (F = 21.932, *P* < 0.001), similar to the comparison between the CA and NCA groups (F = 52.819, *P* < 0.001). Differences in FMD among the CAA subgroups were significant (F = 47.525, *P* < 0.01) (Fig. 3).There were strong negative correlations between these two ultrasonography features only in groups such as the KD(r =  − 0.732, *P* < 0.001), CAA(r =  − 0.935, *P* < 0.001), NCAA(r =  − 0.735, *P* < 0.001), SCA(r =  − 0.845, *P* < 0.001) and CA(r =  − 0.952, *P* < 0.001) groups (Fig. 4). FMD = 3.44% had a sensitivity of 86.1% and specificity of 96.2% in predicting coronary aneurysm formation (area under curve (AUC) = 0.908, 95% confidence interval (95%CI): 0.852–0.965, *P* < 0.001), while FMD = 6.82% (predicting for coronary dilation (preZmax > 2.0)) only had a sensitivity of 63.5% and specificity of 81.3% (AUC = 0.793, 95%CI: 0.707–0.880, *P* < 0.001) (Fig. 5).

**Table 1:** Clinical characteristics and ultrasonography features of KD, Febrile and Healthy groups.

	n	Age*	Male*	BMI*	BBD*	preZmax**
		(years)	n(%)	(kg/m2)	(mm)	
KD	105	2.92 ± 1.86	62(59.0)	16.07 ± 1.35	1.95 ± 0.53	2.57 ± 2.91
Febrile	92	2.77 ± 1.73	51(55.4)	15.99 ± 1.31	2.07 ± 0.54	0.92 ± 1.09
Healthy	79	3.21 ± 1.82	45(57.0)	16.02 ± 1.22	2.04 ± 0.56	0.76 ± 1.12
*P*		0.265	0.876	0.927	0.283	< 0.01

The data are presented as the mean ± SD for continuous variables and as the percentage for the categorical variables. *BMI* body mass index; *BBD* brachial baseline diameter; *preZmax* maximum coronary artery z-score; *FMD* flow-mediated dilation.*P > 0.05(KD v.s. Febrile, KD v.s. Healthy),**P < 0.01(KD v.s. Febrile, KD v.s. Healthy).

## Discussion

FMD is regarded as a reliable ultrasound parameter, which relevantly reflects coronary endothelial function^5,10^. Endothelial dysfunction occurs when FMD is less than 5% as a result of complicated interactions of multitudinous cytokines, molecules, microparticles, etc., which also influence KD-associated vasculitis throughout all phases^11–13^. In this study, we found no significant difference in FMD or preZmax between the febrile and healthy groups, which means common infection-related febrile factors maybe not sufficient to cause neither serious damage to the vascular endothelium nor obvious dilation of the vascular muscularis. On the other hand, the FMD in the KD group was significantly lower than that in both of the other groups, proving that endothelial dysfunction appeared during the acute stage in the majority of KD patients, including those who did not show coronary arterial dilation^14,15^ as a further step based on those studies that considered the corresponding endothelial cells to be damaged to some extent when the coronary artery was dilated by remodelling of the blood vessel wall in the acute phase^11,16–18^. In addition, the FMD in the CAA group in the acute stage was significantly decreased compared with that in the NCAA group, the same between the CA group and the NCA group. FMD significant differences among the mild dilation (MD), SCA, middle coronary aneurysm (MCA) and large coronary aneurysm (LCA) groups. In addition, FMD decreased with increasing coronary artery dilation, suggesting that there may be a correlation between coronary endothelial dysfunction and the degree of coronary artery dilation.

Previous studies have not illustrated how much FMD decreases if there is a clear possibility of coronary artery dilation. To evaluate the dilation more objectively, our study applied preZmax instead of the coronary artery diameter in the analysis of FMD^4,5^. In the following correlation analysis, a strong negative correlation was observed between FMD and preZmax in the KD group, which meant that there might be an increased possibility of abnormal coronary dilation with a decrease in FMD due to the appearance of endothelial dysfunction. In the subgroup comparison, FMD showed a strong negative relationship with preZmax in the CA, CAA and SCA groups, and the absolute value of the correlation coefficient was larger in the CA group than any of the KD, CAA, NA and NCA groups. However, such a relationship was not detected in the MD, MCA or LCA group. Therefore, it is worth mentioning that MD might not be sufficient to impact FMD, which is altered obviously only in the presence of coronary aneurysms. Several studies have shown that the pathological mechanism of mild coronary dilatation in acute phase of Kawasaki disease is different from that of coronary aneurysm, while the former comes before the latter. In the mild dilation stage, the media of affected vessels demonstrate edematous dissociation of the smooth muscle cells, which is most obvious toward the exterior. Endothelial cell swelling and subendothelial edema are seen, but the internal elastic lamina remains intact, so that the inner diameter of the coronary arteries could return to normal in a short time, which maybe a reason for the transient dilation. Destruction of the internal elastic lamina and eventually fibroblastic proliferation occur at the aneurysm stage, after an inflammatory cascade produces endothelial dysfunction and damage to the vascular wall^6,19–24^. According to the pathogenesis and our findings, it might be suggested that kids with worse endothelial dysfunction may have greater chance to get CA in acute phase of KD than those just with mild dilation. However, we couldn't clarify quiet clearly the relationship between Z-score (or inner diameter of coronary artery ) and FMD( or vascular endothelial function) as well as the variation trend of inner diameter further for KD children who already had coronary aneurysms in acute stage, since the number of cases of MCAs and LCAs might be too small to detect a significant difference. The underlying mechanisms of this coronary diameter-related injury in endothelial inflammation require further exploration^15^.

Furthermore, although the relationship in all other CAA subgroups was weaker than that in the NCAA group, the strongest negative relationship among the CAA subgroups was observed in the CA group, meaning that the degree of coronary dilation became sensitive to endothelial dysfunction upon SCA formation or when preZmax ranged from 2.5 to bigger. The above results suggest that simple linearity may be inadequate to explain the relationship between endothelial dysfunction and coronary abnormalities, especially for those with middle and large coronary aneurysms. Further studies with a larger number of MCA and LCA cases on compensatory mechanisms, time course, etc., are needed to determine the exact mathematical relationship between these two indicators. Nevertheless, we still need to emphasize that the dynamic inflammation in endothelial dysfunction may starts mostly at the beginning of SCA development during the progression of KD. From this period on, some irreversible imbalance could have gradually formed in the endothelial immune system^25–27^, which may aggravate coronary expansion^15,28,29^.

In the further determination of the optimal threshold for predicting CAA, we found that FMD ≤ 3.44% had good sensitivity and specificity for SCA and more severe types. It is advantageous to pay attention to the moment of SCA formation or FMD ≤ 3.44% and increase the frequency of monitoring the inner diameter of the coronary artery to provide supportive treatment for vascular endothelial immune reconstruction and regeneration^30–33^, since these measures may have a positive effect on the recovery of damaged coronary endothelial function and the prevention of the further development of an MCA, an LCA or coronary constriction caused by inflammation going deep into the muscularis and coronary vessel wall remodelling^34^. The above speculation may also be supposed as a reference for the administration time in both basic and clinical research on medicines for early KD treatment.

In conclusion, FMD could sensitively indicate the pathological process of acute coronary artery dilation, especially for aneurysms when it is less than 3.44%, by demonstrating arterial endothelial dysfunction at the beginning of KD.

### Limitations

The following limitations to our study should be considered. First, this was a single-centre study that lasted for only two years. Second, we only focused on the acute phase of KD, and the results could be more convincing if the subacute phase, convalescence and even adulthood were all considered. Third, there were only a few cases of MCA and LCA. Selection bias and ascertainment bias could not be ruled out. Further longitudinal studies with a larger number of special cases, with a longer follow-up time and including various phases are recommended.

## Data Availability

The authors confirm that all the data based findings are fully available without restriction. All relevant data are included in the paper and references.

## Abbreviations

AHA: American Heart Association
AUC: Area under the curve
BBD: Brachial baseline diameter
BMI: Body mass index
CAA: Coronary artery abnormality
CA: Coronary aneurysm
CI: Confidence interval
FMD: Flow-mediated dilatation
KD: Kawasaki disease
LAD: Left anterior descending
LCA: Large coronary aneurysm
LCX: Left circumflex artery
LMCA: Left main coronary artery
MCA: Middle coronary aneurysm
MD: Mild dilation
NCAA: No coronary artery abnormality
NCA: No coronary aneurysm
RCA: Right coronary artery
ROC: Receiver operating characteristic
SCA: Small coronary aneurysm
preZmax: Pretreatment maximum coronary artery Z-score
